# Unraveling Liquid–Liquid Phase Separation (LLPS) in Viral Infections to Understand and Treat Viral Diseases

**DOI:** 10.3390/ijms25136981

**Published:** 2024-06-26

**Authors:** Marie Galloux, Sonia Longhi

**Affiliations:** 1 INRAE, Unité de Virologie et Immunologie Moléculaires (VIM), Université Paris-Saclay-Versailles St. Quentin, 78350 Jouy-en-Josas, France; 2 Laboratoire Architecture et Fonction des Macromolécules Biologiques (AFMB), UMR 7257, Aix Marseille University and CNRS, 13288 Marseille, France

In the field of virology, liquid–liquid phase separation (LLPS) has emerged as a pivotal mechanism enabling the compartmentalization required for specific steps of the viral replication cycle. This phenomenon, leading to the formation of liquid-like cytoplasmic or nuclear membraneless organelles (MLOs) in infected cells, has revolutionized our understanding not only of viral replication, but also of pathogenesis and control of host response by viruses. Many viruses, and in particular negative-sense single-stranded RNA viruses (-ssRNA), exploit LLPS for their replication either to form viral factories or to interfere with the (dis)assembly and regulation of host MLOs [1,2,3,4,5]. Viral factories are sites where viral replication and assembly take place and where specific viral and cellular proteins, as well as nucleic acids, concentrate; not only do they serve as hubs for optimized viral replication via the selective uptake or exclusion of cellular components, but they also enable the evasion of the host response through the trapping of cellular proteins of innate immune pathways [6]. Interference with host MLOs take places through interaction with cellular proteins, which leads to the sequestration of cell sensors of pathogen-associated molecular patterns (PAMPs), host cell proteins of the innate immune response, and/or proteins of the integrated stress response, e.g., stress granule proteins. By compartmentalizing essential viral components and hijacking host cell machinery, these liquid-like condensates thus facilitate efficient viral propagation while evading the host immune system. More recently, a cross-talk between membrane-delimited viral replication organelles (VROs), which are typical of positive-sense single-stranded RNA viruses (+ssRNA), and biocondensates resulting from LLPS has been highlighted [7,8], thus further extending the complexity of viral replication compartment morphogenesis and dynamics. 

So far, the majority of studies have focused on LLPS in relation to replication compartments, and relatively few studies have described interference with host functions. Likewise, only a few studies have focused on the functional impact of phase transitions towards gelled and/or fibrillar states of viral condensates. Examples of fibrils made of viral proteins have just begun to be reported, and the possible implications for pathogenesis are just starting to be discussed [9]. 

Exploring the formation, dynamics, and material properties of viral condensates is key to shedding light on fundamental virological processes and will also pave the way towards the potential development of novel therapeutic strategies. 

In the last few years, an increasing number of studies have shown that biomolecular condensates resulting from LLPS are driven by weak and multivalent protein–protein and/or protein–nucleic acid interactions [10,11,12]. Multivalency, i.e., the ability to establish multiple interactions via multiple interaction domains or motifs, is typical of, although not strictly restricted to, intrinsically disordered proteins (IDPs) and regions (IDRs), thus explaining why MLOs are enriched in IDPs/IDRs [13]. LLPS can be further modulated by post-translational modifications (PTMs) [14], with PTM sites being generally enriched in IDPs/IDRs. 

The characterization of the intricate interplay between viral proteins, nucleic acids, and cellular factors in MLOs morphogenesis and dynamics still remains to be elucidated. Unraveling the structural and dynamic properties of viral condensates may unveil novel vulnerabilities that can be exploited for therapeutic purposes, ranging from small-molecule inhibitors to RNA-based therapeutics [15,16,17]. More specifically, targeting key regulators of LLPS, such as viral RNA-binding proteins or host cell chaperones, presents a promising avenue for disrupting viral replication and mitigating disease progression.

It is important to emphasize that the study of LLPS in vitro and in cellula has benefited from huge advances in techniques and approaches, specifically in biophysics and microscopy. However, studying these mechanisms and organelles in the context of viral infections still remains highly challenging. Traditional biochemical and imaging techniques often struggle to capture the transient and heterogeneous nature of these dynamic structures. High-resolution microscopy techniques, such as super-resolution imaging and live-cell imaging, offer glimpses into the spatio-temporal dynamics of viral condensates, but are limited by phototoxicity, resolution, and sample preparation requirements. Additionally, quantitative methods for analyzing LLPS, such as fluorescence recovery after photobleaching (FRAP), Fluorescence Loss Induced by Photobleaching (FLIP), fluorescence correlation spectroscopy (FCS), and single-particle tracking (SPT), require sophisticated instrumentation and data analysis expertise. Finally, cryo-electron tomography (cryo-ET) has emerged as a powerful tool for investigating the structures and dynamics of biomolecular condensates [18]. This technique enables the three-dimensional reconstruction of cellular compartments at nanometer resolution, providing insights into the morphology, composition, and spatial relationships of MLOs formed through LLPS. Overall, it is increasingly recognized that understanding LLPS during infections requires combining different approaches, from molecular to cellular levels (Figure 1). 

In conclusion, unraveling the intricacies of LLPS in viral infections is of paramount importance, with profound implications for both basic science and clinical practice. By dissecting the molecular choreography of viral condensates and overcoming technical hurdles, we can deepen our understanding of viral pathogenesis, identify druggable targets, and accelerate the development of next-generation antiviral therapies. Embracing multidisciplinary approaches will be essential in harnessing the full therapeutic potential of LLPS modulation in combating viral diseases.

This Special Issue collects contributions that cover different aspects of viral condensate formation, including emerging approaches for their quantitative study and new promising antiviral approaches that target these processes. 

## Figures and Tables

**Figure 1:**
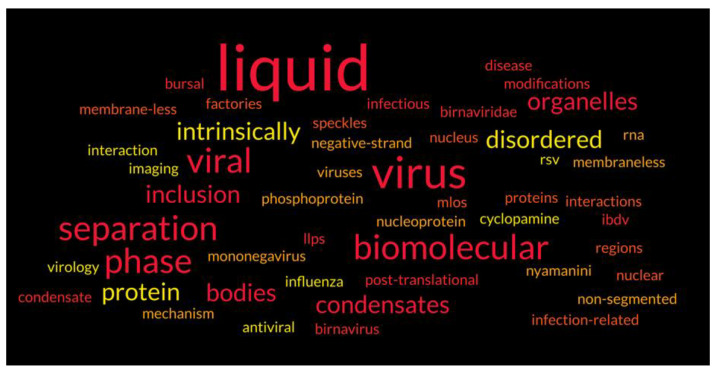
Cloud analysis of key words of papers of the Special Issue on Viral Condensates and Virus Interference with Host Membraneless Organelles.
